# Response kinetics reveal novel features of ageing in murine T cells

**DOI:** 10.1038/s41598-019-42120-1

**Published:** 2019-04-03

**Authors:** Daan K. J. Pieren, Noortje A. M. Smits, Martijn D. B. van de Garde, Teun Guichelaar

**Affiliations:** Centre for Infectious Disease Control, National Institute for Public Health and the Environment, Bilthoven, The Netherlands

## Abstract

The impact of ageing on the immune system results in defects in T cell responsiveness. The search for ageing hallmarks has been challenging due to the complex nature of immune responses in which the kinetics of T cell responsiveness have largely been neglected. We aimed to unravel hallmarks of ageing in the kinetics of the murine T cell response. To this end, we assessed ageing-related T-cell response kinetics by studying the effect of the duration and strength of *in vitro* stimulation on activation, proliferation, and cytokine secretion by T cells of young and aged mice. Collectively, our data show that stimulatory strength and time kinetics of cytokine secretion, activation markers, and proliferation of Th, Tc, and Treg cells are crucial in understanding the impact of ageing on T cells. Despite low proliferative capacity, T cell subsets of aged mice do respond to stimulation by upregulation of activation markers and secretion of cytokines. These findings therefore indicate that replicative senescence of aged T cells is not a measure of unresponsiveness per se, but rather stress that ageing influences the kinetics of proliferation, upregulation of activation markers and cytokine secretion each to a different extent.

## Introduction

The immune system reflects consequences of ageing by many alterations in the T-cell population that compromise T-cell responsiveness at old age^1,2^. Ageing-related changes have been widely reported in helper T cells (Th), cytotoxic T cells (Tc), and regulatory T cells (Treg) that act in concert to provide T cell-mediated immunity. Changes due to ageing occur among a wide variety of different immune parameters, such as the induction of cell surface activation markers, secretion of cytokines, and proliferative capacity^3–6^. The complexity to which ageing alters T-cell responses poses a major challenge in research on T-cell ageing. Whereas many studies address ageing-related T-cell phenotypes, only limited insight is available on the impact of ageing on the response kinetics over time^7^. In this study, we assessed ageing-related T-cell response kinetics by studying the effect of the duration and strength of *in vitro* stimulation on activation, proliferation, and cytokine secretion by T cells of young and aged mice.

T cells of humans and mice rapidly upregulate expression of classical activation markers CD69 and CD25 after stimulation^8,9^. Upregulation of these markers at older age in human and murine T cells is reduced^10–13^. Expression of Programmed cell death-1 (PD-1) and Cytotoxic T-lymphocyte-associated antigen 4 (CTLA-4) is upregulated after T-cell activation^14,15^, yet a substantial proportion of T cells of aged mice show constitutive expression of these inhibitory markers^16–19^. Additionally, ageing diminishes the capacity of T cells to proliferate in humans and mice^7^. Reduced proliferation is a major characteristic of T-cell senescence^6^. As both proliferation and expression of activation and inhibition markers by T-cell subsets are highly dynamic during an immune response, elucidating the kinetics of these parameters may reveal ageing-related alterations of T-cell responsiveness.

Cytokine secretion after *in vitro* stimulation of T cells is also known to alter with ageing in both humans and mice^20^. However, findings are highly ambiguous in part due to the lack of studies addressing time kinetics of cytokine secretion, while these kinetics are vital for understanding ageing-related alterations^20^. For example, many studies in both humans and mice have shown contradicting results on the impact of ageing on IFN-γ^12,16,21–29^ and IL-2^20–23,27,30–35^ secretion by cells. In addition, a suggested shift from a Type-1 towards a Type-2 cytokine secretion profile due to ageing^36,37^ has also been counteracted in other studies^20,21,23,24^. The lack of consensus on the impact of ageing on secreted cytokines may be caused by a lack of time kinetics in cytokine secretion assays as well as differences in strength of stimulation.

In this study, we aimed to reveal the impact of ageing on T-cell responsiveness by assessing the *in vitro* response kinetics of cytokine secretion, activation marker upregulation, and proliferation of T cells of young and aged mice in response to antigen-independent stimulation. We found that despite low proliferative capacity, T cell subsets of aged mice do respond to stimulation by upregulation of activation markers and secretion of cytokines. Furthermore, dimensionality reduction (viSNE)^38^ analyses allowed us to assess the phenotypical changes occurring in T cells over time and revealed increased variation in the responsiveness of T-cell subsets of aged mice. Our findings stress the importance of addressing T-cell response kinetics and the strength of stimuli used to characterise the impact of ageing on the T cell compartment.

## Results

### Proportion of splenic regulatory T cells increases with progressing age

**Figure 1:**
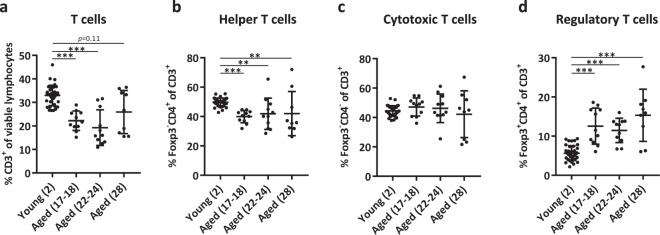
Proportion of regulatory T cells increases with progressing age. The frequencies of T cells (CD3^+^ of viable lymphocytes) (**a**), Th cells (FoxP3^−^ CD4^+^ of CD3^+^) (**b**), Tc cells (FoxP3^−^ CD4^−^ of CD3^+^) (**c**), and Treg cells (Foxp3^+^ CD4^+^ of CD3^+^) (**d**) were determined in spleens of young (2 months old, n = 36) and aged mice of three age categories (17–18 months old, n = 11; 22–24 months old, n = 11; 28 months old, n = 9). Numbers between brackets indicate the age in months. Mean ± SD; **p < 0.01, ***p < 0.001 for difference between young and each group of aged mice using Kruskal-Wallis test.

We assessed the composition of the total splenic CD3^+^ T-cell pool of young (n = 6 per experiment, 2 months old) and aged mice of various ages (n = 4–6 per experiment, 17 to 18 months, 22 to 24 months, and 28 months old). Using flow cytometry, significantly lower frequencies of CD3^+^ T cells were detected in the spleens of aged mice compared to young mice, except for the oldest group of mice (Fig. 1a, Supplementary Fig. 1). Within this T-cell pool, we found decreased proportions of Th cells (CD3^+^CD4^+^FoxP3^−^) (Fig. 1b), comparable proportions of Tc cells (CD3^+^CD4^−^FoxP3^−^) (Fig. 1c), and increased proportions of Treg cells (CD3^+^CD4^+^FoxP3^+^) (Fig. 1d) with progressing age. Defining Tc cells as CD3^+^CD4^−^FoxP3^−^ may also include small proportions of non-Tc-cell subsets, such as CD4^−^CD8^−^ T cells or γδ T cells. However, an additional data set showed that the proportion of these cells did not differ between young and aged mice (Supplementary Fig. 1).

### Proliferative T-cell responses decline with progressing age

**Figure 2:**
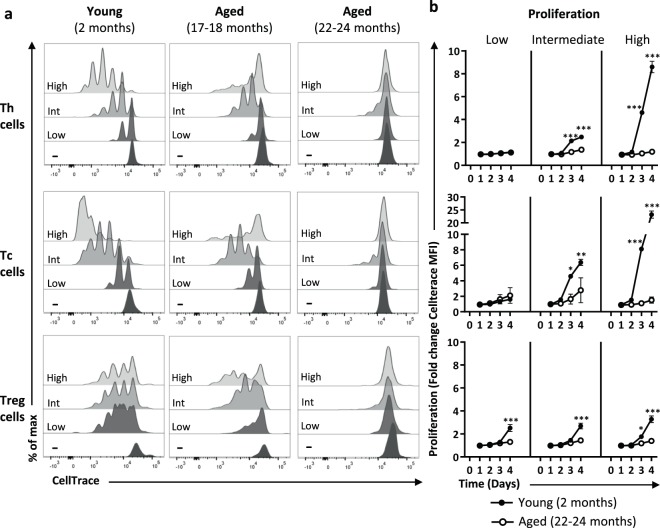
Gradual loss of proliferative capacity in Th, Tc, and Treg cells with ageing. (**a**) The proliferative capacity of Th, Tc, and Treg cells of young (2 months old) and aged (17–18 and 22–24 months old) mice was measured four days after exposure to a low, intermediate, or high stimulatory strength. For each condition graphs show data of one mouse that is representative of n = 6 per age group. (**b**) Graphs show proliferation of Th, Tc, and Treg cells by fold change of proliferation marker CellTrace in young (n = 6, 2 months old) and aged (n = 6, 22–24 months old) mice after four days cultured in the presence of a low, intermediate, or high stimulatory strength. Mean ± SEM; *p < 0.05, **p < 0.01, ***p < 0.001 for difference between young and aged mice using Two-way ANOVA.

Next, we exposed splenic single-cell suspensions of young and aged mice to a low (low anti-CD3), intermediate (low anti-CD3 + anti-CD28), or high (high anti-CD3 + anti-CD28) strength of stimulation to investigate the proliferative capacity of Th, Tc, and Treg cell subsets. Proliferation of Th, Tc, and Treg cells was measured by flow cytometry up to four days after stimulation (Fig. 2). Proliferative capacity of all three T-cell subsets showed a gradual decline with older age (Fig. 2a). Stronger stimulation induced higher rates of proliferation in Th and Tc cells of young mice, which was most pronounced after four days (Fig. 2b). In contrast, the Th and Tc cells of mice of 22 months and older did not show any proliferative response to these stimuli over time. Proliferation of Treg cells of young mice did not increase in response to higher strength of stimulation, but Treg cells of young mice proliferated significantly more compared to aged mice under any condition tested. These data indicate a loss of capacity to proliferate at old age across all T cell subsets that cannot be overcome by increasing the stimulatory strength.

### Ageing-related alterations in cytokine profiles depend on response kinetics and strength of stimulation

**Figure 3:**
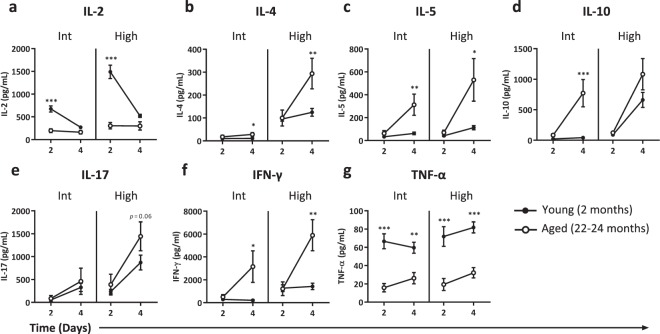
Duration and strength of stimulation determine ageing-related cytokine profiles. Cytokine levels of IL-2 (**a**), IL-4 (**b**), IL-5 (**c**), IL-10 (**d**), IL-17 (**e**), IFN-γ (**f**), and TNF-α (**g**) were measured in supernatant of splenocytes exposed for two or four days to an intermediate (int) or high stimulatory strength. Data represent one experiment with young (n = 6, 2 months old) and aged (n = 6, 22–24 months old) mice. Mean ± SEM; *p < 0.05, **p < 0.01, ***p < 0.001 for difference between young and old mice at time points indicated using Two-way ANOVA.

Cytokine production by stimulated T cells changes with ageing, which has widely been shown by measurements of cytokines secreted in supernatants. However, such findings on are highly ambiguous due to the lack of studying the kinetics of cytokine responses, as studies often address cytokine secretion with a single stimulatory dose and at one point in time^20^. We examined the impact of ageing on the induction of effector cytokines during the response of T cells to differing stimulatory strengths after two and four days of culturing (Fig. 3).

Stimulation of T cells with a low concentration of anti-CD3 did not result in detectable cytokine levels after two and four days in both young and old mice (data not shown). Using intermediate or high strength of stimulation, cells of young mice produced significantly higher levels of IL-2 at day two compared to cells of aged mice (Fig. 3a). Cells of aged mice showed stronger Th2-related responses, with significantly higher IL-4 (Fig. 3b) and IL-5 (Fig. 3c) production compared to cells of young mice four days after exposure to intermediate or high strength stimuli. In addition, secretion of IL-10 was found only at day four, and this cytokine was produced at higher levels by cells of aged mice (Fig. 3d). Interestingly, cells of aged mice but not young mice, produced high amounts of IL-10 in response to intermediate strength of stimulation, which indicates that aged mice require less stimulation to trigger IL-10 production compared to young mice.

Detection of ageing-related differences in secretion of pro-inflammatory cytokines IL-17, IFN-γ, and TNF-α also depended on the time and strength of stimulation. Aged mice showed a trend towards higher IL-17 secretion, but only after four days and by high strength stimulation (Fig. 3e). Aged mice also showed higher IFN-γ secretion, which became apparent only after four days of stimulation with both intermediate and high strength stimulation (Fig. 3f). TNF-α secretion by cells of aged mice was consistently lower compared to secretion by cells of young mice (Fig. 3g). Taken together, these results indicate that both time and the stimulatory strength are important in assessing ageing-related cytokine profiles.

Measuring cytokines in supernatant does not indicate which T cell subset accounts for the cytokines produced. Therefore, we investigated the maximum potential of CD4^+^ and CD8^+^ cells to intracellularly express Th1-related (IFN-γ and TNF-α) and Th2-related (IL-4 and IL-5) cytokines during a 4 hour stimulation with PMA/ionomycin (Supplementary Fig. 2). Aged mice showed significantly higher frequencies of IFN-γ-producing cells in both CD4^+^ and CD8^+^ T cells, suggesting that Th and Tc cells both contribute to the higher IFN-γ we measured in supernatants (Fig. 3f). In contrast to higher TNF-α in the supernatant (Fig. 3g), CD4^+^ and CD8^+^ T cells of aged mice produced higher levels of TNF-α than those of young mice. This difference may be due to the different modes of stimulation or by different rates of TNF-α consumption. Additionally, the higher levels of IL-4 and IL-5 we detected in the supernatant of aged mice was paralleled by a significantly higher frequency of aged IL-4/IL-5^+^ CD4^+^ T cells (Supplementary Fig. 2). This suggests that predominantly CD4^+^ T cells account for the elevated levels of IL-4/5 found in supernatants of spleen cells cultured from aged mice.

### Reduced induction of T cell activation in aged mice can partially be restored by stronger stimulation

**Figure 4:**
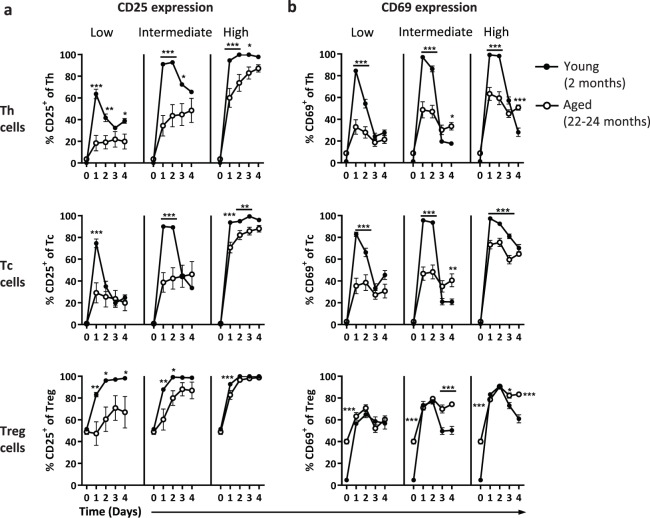
Reduced induction of T cell activation in aged mice can partially be restored by stronger stimulation. The proportion of CD25^+^ (**a**) and CD69^+^ (**b**) Th, Treg, and Tc cells of young (n = 6, 2 months old) and aged (n = 6, 22–24 months old) mice cultured up to four days with a low, intermediate, or high stimulatory strength. Mean ± SEM; *p < 0.05, **p < 0.01, ***p < 0.001 for difference between young and aged mice at time points indicated using Two-way ANOVA.

We next analysed the expression of the classical activation markers CD69 and CD25 to investigate to what extent ageing influences the activation kinetics of T cells (Fig. 4). After low strength stimulation, the frequency of CD25^+^ Th and Tc cells of young mice increased and peaked at day one (Fig. 4a). With higher strength of stimulation, the maximum frequencies of CD25^+^ cells induced were higher and maintained over time, irrespective of age. In Th and Tc cells of aged mice, maximum frequencies of CD25^+^ cells however never reached those of young mice. Treg cells of aged mice showed significantly lower frequencies of CD25^+^ cells compared to young mice in response to both low and intermediate strength of stimulation. However, high dose stimulation resulted in comparable CD25^+^ Treg frequencies between young and aged mice (Fig. 4a), although the expression of CD25 per cell was lower on aged Treg cells (data not shown).

Frequencies of cells expressing the early activation marker CD69 on Th and Tc cells were comparable between young and aged mice before stimulation, while the frequency of CD69^+^ Treg cells of aged mice was significantly higher (Fig. 4b). At young age, all three T-cell subsets showed a rapid incline in the frequency of CD69^+^ cells within one day. At old age, Th and Tc cells also quickly upregulated CD69 with a stimulatory dose-dependent increase, but never reached the maximum CD69^+^ cell frequencies of young cells. In contrast, Treg cells of old mice expressed similar or higher frequencies of CD69^+^ cells than Tregs of young mice after stimulation. Moreover, after one to two days of culture, all three subsets of young mice showed a decline in the frequency of CD69^+^ cells. This decline was less pronounced on the T cells of aged mice, as shown by persistent CD69^+^ cell frequencies, leaving stimulation-induced frequencies of CD69^+^ cells observed at later time points of the culture higher in the aged mice than in the young mice. Thus, these data indicate that activation kinetics of T cells at old age can in part be improved by increasing the stimulatory strength. However, a substantial fraction of the Th and Tc cells of aged mice still refrain from expressing CD25 or CD69.

### T cells of aged mice do not proliferate in response to exogenous IL-2

T cells of aged mice showed diminished proliferation, lower IL-2 secretion, and altered CD25 expression kinetics in response to stimulation (Figs 2–4). As IL-2 is an important driver of T cell proliferation, we hypothesized that the deficiency of IL-2 in culture may contribute to the diminished T cell proliferation observed in aged mice. Therefore, we assessed whether exogenous IL-2 could overcome the IL-2 deficiency and partially restore defective proliferation of T cells at old age. We stimulated cells of young and aged mice with a low stimulatory strength of anti-CD3+/− exogenous recombinant murine IL-2 and monitored Th, Tc, and Treg cell proliferation (Supplementary Fig. 3). Addition of exogenous IL-2 did not enhance proliferation in T cell subsets of aged mice, whereas it enhanced proliferation of Th and Tc cells of young mice (Supplementary Fig. 3).

### Ageing influences naive and memory CD4^+^ and CD4^−^ T-cell heterogeneity by enrichment for regulatory cell types

**Figure 5:**
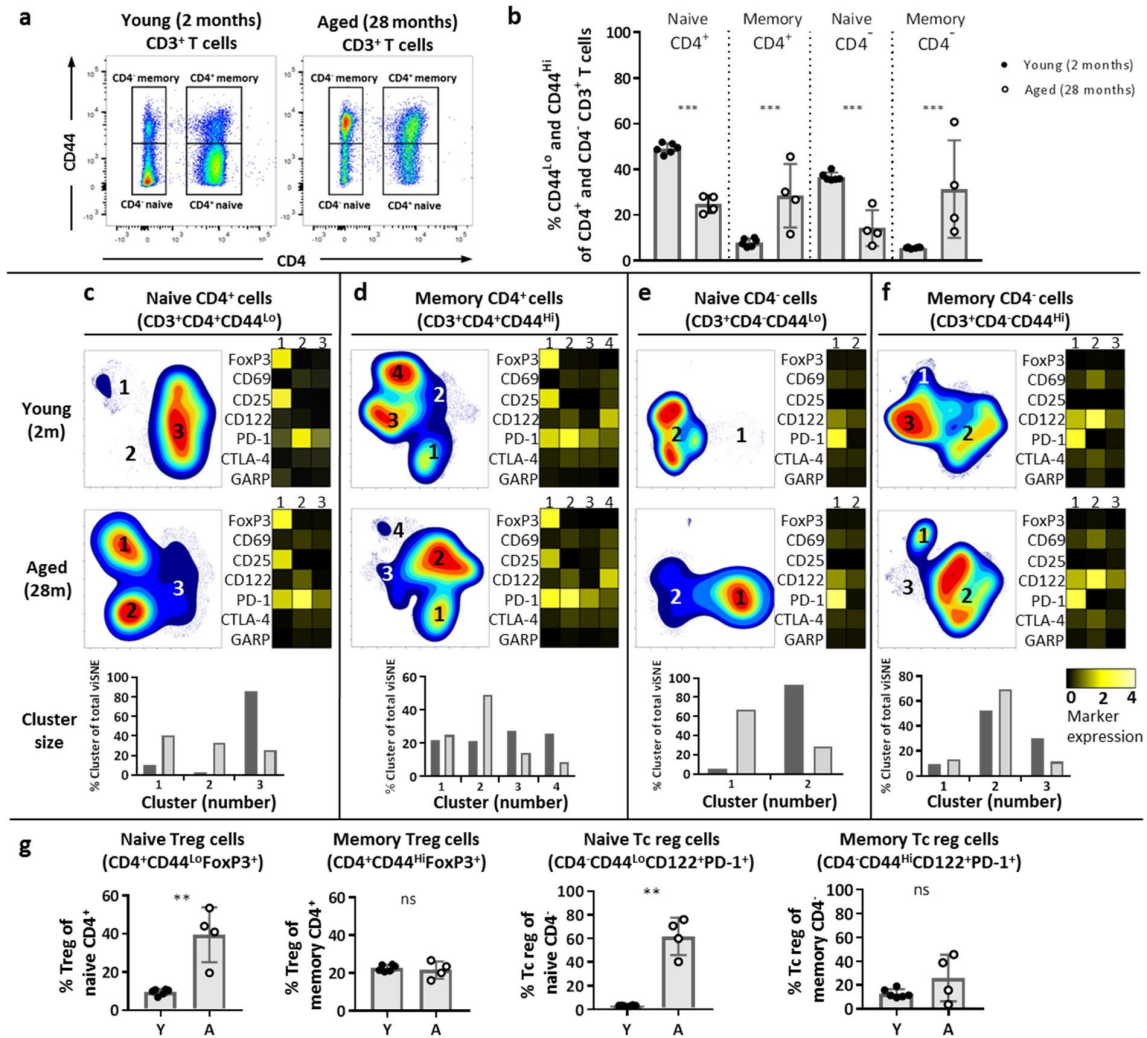
Aging impacts the heterogeneity found within naive and memory T cells and enriches the naive compartment with cells expressing a Treg phenotype. Naive (CD44^Lo^) and memory (CD44^Hi^) cells were identified in the CD4^+^ and CD4^−^ T populations by expression of CD44 (**a**). Frequencies of naive and memory cells in the CD4^+^ and CD4^−^ T cell populations were determined in young (n = 6, 2 months old) and aged (n = 4, 28 months old) mice (**b**). Cell density maps of dimensionality reduced single-cell data show clustering within either naive or memory populations of CD4^+^ (**c**,**d**) and CD4^−^ T cells (**e**,**f**) from pooled samples of young or aged mice. Numbers in the clusters on the density maps correspond to the cluster numbers in the heatmaps shown to the right of each cell density plot (**c**–**f**). Bar graphs below the density and heatmaps indicate the proportions of each cluster defined within the total viSNE (**c**–**f**). Heatmaps depict the ArcSinh5-transformed median expression of the designated markers of the respective clusters. The frequency of Treg cells of CD4^+^ naive and memory cells (FoxP3^+^ of CD3^+^CD4^+^CD44^Lo/Hi^) and Tc reg cells of CD4^−^ naive and memory cells (PD-1^+^ CD122^+^ of CD3^+^CD4^−^CD44^Lo/Hi^) was analysed by flow cytometry in young (Y) and aged (A) mice (**g**). Mean ± SD; **p < 0.01, ***p < 0.001 for difference between young and aged mice using Mann-Whitney test.

Age-related differences found in T cell responses have largely been ascribed to the shifted balance of naive T cells towards memory T cells during ageing rather than an ageing-related effect within these cell subsets^2^. We and others observed changes in the composition of the T cell pool at old age other than only a shifted naive/memory balance, such as increase of the Treg cell frequency^39^ (Fig. 1d). We therefore assessed if the impact of ageing may be reflected beyond the mere shift in balance of naive/memory T cells and may be further defined by changes of the heterogeneity within naive and memory T cell populations. To address this question, we first investigated the proportion of naive and memory cells in young and aged mice based on expression of CD44 (Fig. 5a). Aged mice indeed showed decreased naive and increased memory proportions in the CD4^+^ and CD4^−^ T-cell compartments (Fig. 5b).

We next aimed to assess T-cell heterogeneity within naive and memory T-cell subsets. We characterised phenotypically distinct clusters of T cells within the naive and memory cells among the CD4^+^ and CD4^−^ compartments of the T cell pool by application of dimensionality reduction (viSNE) (Supplementary Fig. 4). This panel included the ageing-related markers PD-1 and CTLA-4, as well as the activation markers CD25, CD69, CD122, and Glycoprotein A repetitions predominant (GARP).

Cluster analysis of CD4^+^ and CD4^−^ cells revealed heterogeneity within naive and memory cells, which differed between young and aged mice (Fig. 5c–f). FoxP3, PD-1, CD25, and CD122 accounted for the heterogeneity within these subsets. Cluster 1 of naive and memory CD4^+^ T cells shows high expression of FoxP3, indicating that this cluster is highly enriched for Treg cells (Fig. 5c,d). The proportion of cluster 1 in naive CD4^+^ T cells is larger in aged mice (40.9%) compared to young mice (10.4%) (Fig. 5c), while in memory CD4^+^ T cells the proportion of cluster 1 is comparable between young (22.0%) and aged mice (24.9%) (Fig. 5d). The increased proportion of CD4^+^ Treg cells among the naive CD4^+^ cells is reflected in individual aged mice (Fig. 5g), while there is no ageing-related difference in Treg cell proportions among the memory T cell pool.

Previous studies have described a population of regulatory Tc cells (Tc reg cells) expressing CD122 and PD-1^40,41^. Interestingly, cluster 1 of naive CD4^−^ Tc cells is CD122^+^ PD-1^+^ with a higher proportion of this cluster in the pool of aged mice (Fig. 5e), which is reflected in individual mice (Fig. 5g, Supplementary Fig. 5). The proportion of these Tc reg cells among the memory CD4^−^ pool is comparable between young and aged mice (Fig. 5f,g).

Finally, expression of PD-1 contributed to heterogeneity within naive and memory cell subsets (Fig. 5c–f). This heterogeneity differed with age as aged mice showed higher frequencies of PD-1^+^ in naive Th, Tc, and Treg cells, and in memory Th and Treg cell subsets compared to young mice (Supplementary Fig. 6). Additionally, aged mice showed lower frequencies of CD25^+^ naive Treg cells and lower frequencies of GARP^+^ naive and memory Treg cells (Supplementary Fig. 6).

Thus, naive and memory T cells both are highly heterogeneous and this heterogeneity is changed by ageing. Importantly, ageing influences constitution of these subsets by increasing the expression of PD-1 and by enriching naive CD4^+^ and CD4^−^ cell subsets with cells expressing a regulatory phenotype. Therefore, ageing-related changes may be found beyond the shift from naive towards memory cell pools.

### Increased variation in the responsiveness of T-cell subsets of aged mice

**Figure 6:**
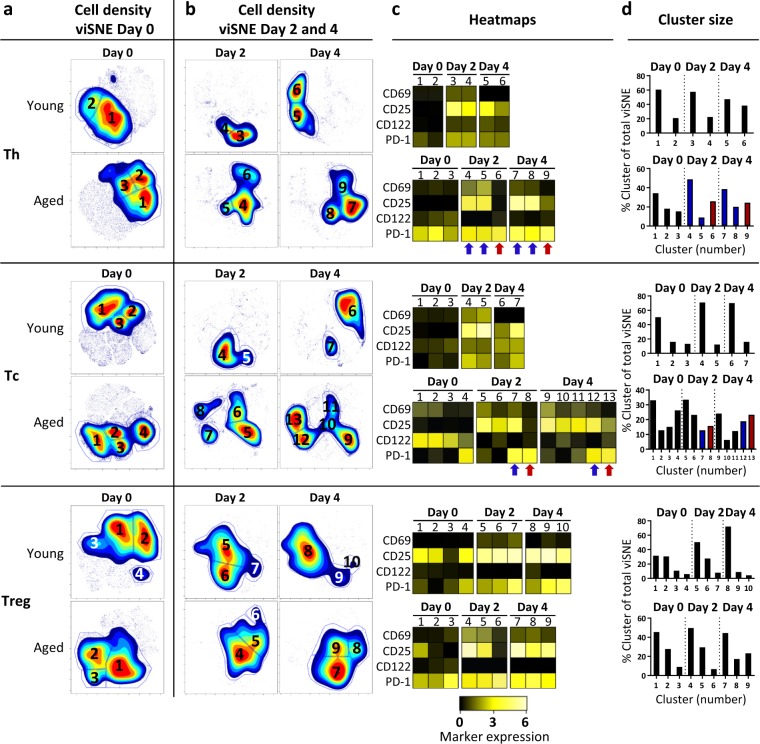
Specific T-cell fingerprints show phenotypical alterations associated with ageing. Splenocytes of young (n = 6, 2 months old) and aged mice (n = 4, 28 months old) were analysed using flow cytometry before and after stimulation for up to four days with an intermediate stimulatory strength. Cell density maps of dimensionally reduced single-cell data show clustering within either Th, Tc, or Treg cell populations of the pooled young and aged mice before stimulation (Day 0) (**a**), and cell density maps at day 2 and 4 after stimulation (**b**). Numbers within the gates correspond to the cluster numbers in the heatmaps (**c**) and bar graphs (**d**). Heatmaps depict the ArcSinh5-transformed median expression of the designated markers in Th, Tc, and Treg cell clusters at the indicated days of young and aged mice. Bar graphs indicate the cell proportions of the clusters identified by viSNE analysis within each T cell subset. Red arrows and bars indicate the non-activated (CD25^−^CD69^−^) PD-1^+^ Th and Tc cell clusters in aged mice (**c,d**). Blue arrows and bars indicate the activated (CD25^+^CD69^+^) PD-1^+^ Th and Tc cell clusters in aged mice (**c,d**).

We next investigated the phenotypical changes that occur within Th, Tc, and Treg cell subsets after exposure to intermediate strength of stimulation (Fig. 6a,b). viSNE analysis of Th, Tc, and Treg cell subsets before stimulation indicated ageing-related differences similar to analyses presented in Fig. 5: Cells of aged mice showed increased expression of PD-1, presence of CD122^+^PD-1^+^ Tc cells, and diminished CD25 expression by Tregs (Day 0, Fig. 6a,c,d, Supplementary Fig. 7).

After stimulation, the number of phenotypically distinct clusters identified in Th cells and Tc cells was higher among cells from aged mice (six Th cell clusters, nine Tc cell clusters) compared to the number of clusters found in Th cells and Tc cells from young mice (four Th clusters, four Tc clusters) (Fig. 6b). The number of Treg cell clusters was comparable between young and aged mice (six clusters) (Fig. 6b).

Differences within each T cell subset of young mice were mainly found in the expression levels of CD69, CD25, CD122, PD-1, and CTLA-4 after stimulation (Fig. 6c, Supplementary Fig. 7). Th, Tc, and Treg cells of young mice showed activation-induced PD-1 and CD25 expression which remained present over time, while their CTLA-4, GARP, CD69 expression was high at day two and had declined by day four (Fig. 6c, Supplementary Fig. 7).

Compared to young mice, Th and Tc clusters of aged mice showed greater phenotypical diversity. Almost all Tc cells of aged mice constitutively expressed CD122 but lost this expression after stimulation, while CD122 on Tc cells of young mice increased over time after stimulation. Moreover, among both Th and Tc-cell subsets, distinct activated (CD25^+^CD69^+^) and non-activated (CD25^−^CD69^−^) clusters were identified. Distinct activated cell clusters (CD25^+^CD69^+^) expressing PD-1 were identified within both the Th and Tc subsets of aged mice after stimulation (Th and Tc, Fig. 6c, blue arrows; Fig. 6d, blue bars). In contrast, also CD25^−^CD69^−^ clusters of PD-1^+^ Th and Tc cells were present (Th and Tc, Fig. 6c, red arrows; Fig. 6d, red bars). For Treg cells, aged mice also showed a cluster of CD25^−^CD69^−^ cells expressing PD-1 after two days of stimulation, but this cluster was not identified at day four (Treg, Fig. 6c).

These data demonstrate that the group of aged mice shows more variation in response to stimulation compared to the group of young mice. These differences are not likely to be due to differences in abundance of naive and memory cells, as PD-1 expression increased with age within both these compartments.

### PD-1^+^ Th and Tc populations contain less activated cells at old age

**Figure 7:**
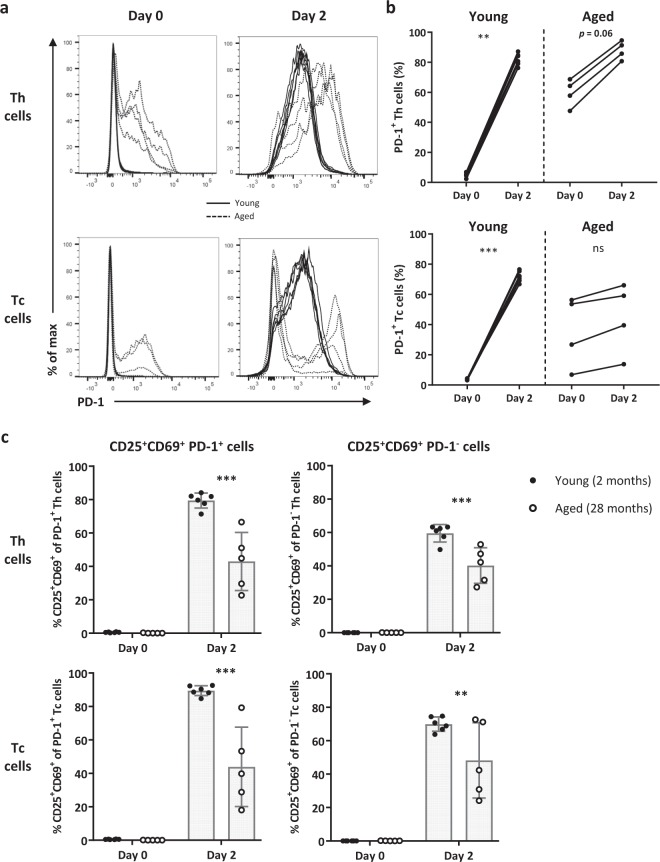
PD-1^+^ Th and Tc populations contain less activated cells at old age. (**a**) Histograms depict expression of PD-1 by Th and Tc cells of young (n = 6, 2 months old) and aged (n = 4, 28 months old) mice at day 0 and at day 2 after culturing with an intermediate stimulatory strength. (**b**) Graphs show the induction of PD-1^+^ Th and Tc cells of young and aged mice after stimulation (day 2). (**c**) The activation status of PD-1^+^ and PD-1^−^ Th and Tc cells from young and aged mice was analysed before and after stimulation. The proportion of activated cells was characterised by the simultaneous expression of CD25 and CD69. Mean ± SD; **p < 0.01, ***p < 0.001 for difference between young and aged mice using Kruskal-Wallis test.

To verify the findings of our viSNE analyses on an individual mouse level rather than a pooled group of mice, we addressed the frequency of activated (CD25^+^CD69^+^) PD-1^+^ and PD-1^−^ Th and Tc cells in young and aged mice before and after two days of exposure to an intermediate strength of stimulation (Fig. 7a,b, Supplementary Fig. 8). The frequency of PD-1^+^ Th and Tc cells of young mice significantly increased after stimulation, whereas cells of aged mice did not (Fig. 7b). Th and Tc cells of aged mice have increased expression of CD25 and CD69 within both PD-1^+^ and PD-1^−^ subsets observed at time points indicated, but to a lesser extent than cells of young mice (Fig. 7c).

## Discussion

In this study, we investigated the impact of ageing on T-cell response kinetics by studying the effect of the duration and strength of *in vitro* stimulation on T-cell activation markers, proliferation, and cytokine secretion in young and aged mice. Our study shows that T-cell response kinetics are a valuable tool to better understand the impact of ageing on T cells. Moreover, our findings indicate that PD-1 expression and impaired proliferation at old age may not imply unresponsive T cells per se.

Decreased induction of CD69 and CD25 expression has been used as a measure for lower rate of activation in CD4^+^ and CD8^+^ T cells of aged humans and mice^7,10–12^, but only few studies have addressed CD25 and CD69 expression kinetics^7^. The stimulation-induced frequency of CD25^+^ and CD69^+^ Th and Tc cells was lower in aged mice compared to young mice during the early phase of the response. Levels found in T cells of aged mice eventually reached levels close to those found in young mice. Moreover, the presence of CD69^+^ cells was more persistent in all T cell subsets of aged mice after their stimulation and became even higher than the declining number of CD69^+^ cells in the T cell pool of young mice at later time points. These findings indicate that early activation of Th and Tc cells of aged mice is diminished and that subsequent downregulation of CD69 is delayed. Persistent CD69^+^ Tc cell frequencies after stimulation have previously been reported in aged mice^7^, and we now show that Th and Treg cell subsets of aged mice also contain cells with persistent stimulation-induced CD69 expression. Persistent frequencies of CD69^+^ Tregs in aged mice may harbour a Treg subset with enhanced suppressor activity^42,43^. Whether such CD69^+^ Tregs may account for reduced Th and Tc cell activation at old age remains to be addressed in future studies.

Treg cells of aged mice could still be adequately activated as they were capable of reaching CD25^+^ cell frequencies similar to those observed in young mice. However, these comparable levels only occurred after increasing the stimulatory strength. This finding may explain earlier reports showing unaltered suppressive functionality of Treg cells at old age despite their reduced CD25 expression at baseline^39,44,45^. Thus, ageing has a differential impact on CD25 kinetics of Treg cells compared to Th and Tc cells.

We observed declined proliferative capacity of Th, Tc, and Tregs cells with progressing age, and increasing the stimulatory strength did not resolve diminished proliferation. Declined proliferation of T cells is a major hallmark of replicative senescence^6^. Moreover, expression of PD-1 has been postulated as a marker of cell populations with diminished proliferative capacity^16,46^. We here showed that none of the PD-1^+^ or PD-1^−^ Tc cells of aged mice older than 24 months proliferated in response to stimulation. Since PD-1^−^ Tc cells do not proliferate at old age, the expression of PD-1 cannot be used as sole predictor for diminished proliferation of Tc cells. Moreover, replicative senescence does not imply that T cell subsets of aged mice are unresponsive, as T cells of aged mice did show upregulation of activation markers and cytokine secretion.

Reports on the impact of ageing on secretion of cytokines are highly ambiguous due to the diversity in stimuli used and the lack of measurements on cytokine response kinetics^20^. Our data highlight the importance of studying both the time and strength of stimulation to address cytokine production. In addition, a shift from a Type-1 to a Type-2 cytokine response with ageing has been reported, but is still under debate^20,21,23,24,36,37^. Our data show no prominent shift from a Type-1 to a Type-2 cytokine profile as the Type-2 cytokines IL-4, IL-5, IL-10, but also Type-1 cytokine IFN-γ were more abundantly secreted by cells from aged mice compared to young mice after four days of culturing. Collectively, our data indicate that aged mice require lower strength of stimulation compared to young mice to produce Type-1 and Type-2 cytokines. This was found in supernatant for most cytokines, and our analyses of intracellular cytokines suggested increased numbers of T cells being able to produce both Th1- and Th2-related cytokines.

In addition to its role as an exhaustion marker, PD-1 can also be considered as an activation marker since it can be induced upon stimulation^16,46,47^. Indeed, in T cells from young mice we found significant upregulation of PD-1 upon stimulation. In contrast to young mice, the frequency of PD-1^+^ cells did not increase in Tc cells of aged mice upon stimulation, which indicates that PD-1 on Tc cells may not be activation-induced at old age. Moreover, in parallel to the absence of significantly increased PD-1 expression upon stimulation, Tc cells did show induced expression of CD25 and CD69. Therefore, these findings may suggest that induced expression of CD69 and CD25 we found on PD-1^+^ Tc cells of aged mice represent activated PD-1^+^ Tc cells. The limited number of cells we obtained from our mice were all used for the assays presented and did not allow extensive sorting of different subsets. Conclusive evidence for the rate of activation of PD-1^+^ versus PD-1^−^ cells upon stimulation requires stimulation of sorted PD-1^+^ and PD-1^−^ Tc cells to provide insight into whether PD-1^+^ cells can be activated.

Age-related differences found in T cells have largely been ascribed to the shifted balance of naive T cells towards memory T cells during ageing^2^. Therefore, many of the aging-related differences have been explained by differences between naïve versus memory cells rather than to aging-related differences within naive or memory T cell populations. We indeed found inflation of the frequency of memory cells by aging. However, analysis of naive and the memory T cell pools revealed that attributing ageing-related differences to different T cell subsets should go beyond mere abundance of naive versus memory cells. Our phenotypic analyses show that the heterogeneity of both the naive and memory pools change with age. Among this heterogeneity, our *ex vivo* multi-dimensional analyses indicate that the naive CD4^+^ and Tc cell pools of aged mice are enriched for cells with a regulatory phenotype. CD122^+^PD-1^+^ Tc cells have been reported to be Tc reg cells^40,41^. The high proportion of naive CD122^+^PD-1^+^ Tc cells observed in aged mice indicates an accumulation of this regulatory cell type with ageing, which has, to the best of our knowledge, not been reported earlier. Additionally, the naive CD4^+^ T cell pool of aged mice was enriched for Foxp3^+^ Treg cells. In addition, aged Treg cells have previously been shown to contain higher proportions of PD-1^+^ cells. We now show that these PD-1^+^ Treg cells are present in both naive and memory Treg cell subsets. Moreover, higher proportions of CD25^low^ Treg cells at old age have been reported^39^, and our data show that these cells are primarily present in the naive Treg cell subset. Our data analysis of combined expression of PD-1 and CD25 indicates a Treg subpopulation of PD-1^+^CD25^Low^ CD4^+^ Tregs that increases with ageing.

T cells at old age showed reduced IL-2 production and lack of proliferation. Since IL-2 is known for its capacity to promote T cell proliferation, this finding suggested that the lack of IL-2 production may contribute to the lack of proliferation we observed at old age. However, addition of exogenous IL-2 did not result in restoration of T cell proliferation in aged mice, indicating that reduced production of IL-2 is not likely to contribute to the reduced proliferation found at old age. Hence, explanations for the defective responsiveness should be searched for in other factors, such as the distorted expression of IL-2 receptor chains CD25 and CD122^9,48,49^ or increased co-inhibitory signals and increased Treg populations we observed at old age. Furthermore, aging-related mediators produced by non-T cells that may function as antigen presenting cells present in our cultures may have contributed to the aging-related defects of T cell proliferation and activation we observed. Although altered co-stimulation by antigen-presenting cells reported with age may interfere with our findings^2,50^, polyclonal stimulation of T cells may largely overrule aging-related effects on APC-mediated co-stimulation and antigen presentation in our assays.

The strength of our analyses lies at examining the T cell pool as a whole within its natural cell composition, which reflects the overall response of all splenocyte subpopulations. Many studies separate CD4^+^ and CD8^+^ naive and memory cells^2,21^. Based on our study, it would be a contribution to the field of ageing for future studies to reveal how purified Th, Tc, and Treg cell subsets respond separately, as well as naive and memory cells within these cell subsets.

Collectively, our data show that stimulatory strength and time kinetics of cytokine secretion, activation markers, and proliferation of Th, Tc, and Treg cells are crucial in understanding the impact of ageing on T cells. Despite low proliferative capacity, T cell subsets of aged mice do respond to stimulation by upregulation of activation markers and secretion of cytokines. These findings therefore indicate that replicative senescence of aged T cells is not a measure of unresponsiveness per se, but rather stress that ageing influences the kinetics of proliferation, upregulation of activation markers and cytokine secretion each to a different extent. Moreover, our multi-dimensional analyses revealed phenotypical T-cell subpopulations that further delineate the impact of ageing on T cells.

## Materials and Methods

### Mice

Young (2 months of age) and aged (age range groups: 17–18 months, 22–24 months, and 28 months of age) C57BL/6 mice were purchased from Envigo (Venray, Limburg, The Netherlands). Mice were maintained at the animal facilities of the Institute for Translational Vaccinology (Bilthoven, Utrecht, The Netherlands).

### Ethics

Animal studies were approved by the Animal Ethical Committee of the National Institute for Public Health and the Environment (DEC no. 201400042). All procedures were carried out in accordance with Dutch national legislation.

### Preparation of single cell suspensions and proliferation labelling

Spleen single cell suspensions were prepared by homogenizing the spleen through a cell strainer. Red blood cells were lysed with ACK lysis buffer (0.155 M NH4Cl; 10 mM KHCO3; 0.1 mM Na2EDTA, pH 7.2–7.4). Labelling to track proliferation was performed as follows: splenocytes were resuspended in PBS to 10*10^6^ cells/mL and then labelled with 0.5 μM CellTrace^TM^ Violet (Invitrogen, Carlsbad, CA, USA) in PBS per millilitre of splenocyte suspension for 20 minutes at 37 °C. Cells were then washed twice with ice-cold RPMI (Gibco, Thermo Fisher Scientific, Waltham, MA, USA) medium containing 10% fetal calf serum (FCS, Greiner Bio-One, Kremsmünster, Austria).

### *In vitro* antigen-independent stimulation and splenocyte culture

Soluble anti-CD3 (clone 145-2C11, eBioscience, San Diego, CA, USA) and anti-CD28 (aCD28, clone PV-1, SouthernBiotech, Birmingham, AL, USA) were used to stimulate cells to a low (0.019 μg/mL anti-CD3), intermediate (0.019 μg/mL anti-CD3 + 0.5 μg/mL anti-CD28), or high (0.5 μg/mL anti-CD3 + 0.5 μg/mL anti-CD28) extent over time. For proliferation assays in response to exogenous IL-2, 0.1 μg/mL recombinant murine IL-2 (eBioscience) was added with and without the presence of low anti-CD3 (0.019 μg/mL). Stimulatory conditions were prepared in RPMI medium containing 10% FCS and then added to the splenocyte suspensions (4*10^5^ cells/well) before incubation in 96-well U-bottom plates (CELLSTAR, Greiner bio-One) at 37 °C for up to four days.

### Immunofluorescence labelling and flow cytometric analyses

Single cell suspensions were washed and labelled at 4 °C for a combination of cell surface markers with the following antibodies: anti-CD4-BUV395 (clone GK1.5), anti-CD8a-V450 (clone 53–6.7), anti-CD122-PE-CF594 (clone TMbeta1), anti-CD44-V450 (clone IM7) and anti-CD69-BV786 (clone H1.2F3) (BD Horizon, Franklin Lakes, NJ, USA); anti-CD69-PerCP-Cy5.5 (clone H1.2F3) and anti-PD-1-BV785 (clone 29F.1A12) (BioLegend, San Diego, CA, USA); anti-CD25-PECy7 (clone PC61.5) (eBioscience); and Live/Dead Fixable Aqua (Invitrogen). Cells were labelled intracellularly with the following antibodies according to the FoxP3 Transcription Factor staining buffer set protocol (eBioscience): anti-CD3zeta-FITC (clone H146-968) (Abcam, Cambridge, Cambridgeshire, UK); anti-CTLA-4-BV605 (clone UC10-4B9), and anti-TNF-α-BV785 (clone MP6-XT22) (BioLegend); anti-FoxP3-eFluor660 (clone 150D/E4), anti-GARP-PE (clone YGIC86), anti-IFN-γ-PE-Cy7 (clone XMG1.2), and IL-4-PE (clone 11B11) (eBioscience); anti-IL-5-PE (clone TRFK5) (BD Pharmingen). Labelled cells were detected on a BD LSRFortessa X-20 (BD Biosciences, Franklin Lakes, NJ, USA). Gating analyses were performed using FlowJo software (Tree Star, Ashland, OR, USA).

### Cell proliferation

Proliferation of cells was measured using detection of CellTrace by flow cytometry. As reported earlier^51^, the fold change proliferation was calculated by dividing the CellTrace median fluorescent intensity (MFI) of the medium control by the stimulated cells of each individual animal.

### Dimensionality reduced analyses

Dimensionality reduced analyses (viSNE) of flow cytometry data were performed in Cytobank (www.cytobank.com)^38^. Cell density maps of dimensionally reduced single-cell viSNE data showing clustering of CD4^+^ and CD4^−^ naive and memory populations or Th, Tc, and Treg-cell subsets of six pooled young mice (2 months old) and four pooled aged mice (28 months old) before and after receiving an intermediate strength of stimulation for four days. Expression of the designated cellular markers in the heatmaps was based on their ArcSinh5-transformed median expression. Indicated cluster frequencies were based on the cells present within the gates of the viSNE plots. The total viSNE plot of each T cell subset for each indicated day comprised 100% of cells of that subset.

### Cytokine assays in supernatant

Supernatants of cell cultures were stored at −80 °C and thawed before measuring the cytokines present. Secreted cytokines in culture supernatants were measured by using a Milliplex MAP kit Mouse Cytokine/Chemokine Magnetic Bead Panel (Millipore, Burlington, MA, USA) according to the manufacturer’s instructions. The following cytokines were measured: IL-2, IL-4, IL-5, IL-10, IL-17, IFN-γ, and TNF-α. Cytokines were detected on a Luminex (Bio-Rad, Hercules, CA, USA).

### Intracellular cytokine analysis

For intracellular cytokine labelling, splenocyte suspensions (2*10^5^ cells/well) of young and aged mice were cultured with phorbol 12-myristate 13-acetate (PMA) (0.05 μg/mL) and ionomycin (0.5 μg/mL) (Sigma Aldrich) in medium for four hours in total. After one hour of culturing, GolgiPlug (containing Brefeldin A; 1:1000, BD Biosciences) was added to allow intracellular accumulation of produced cytokines. After labelling with anti-CD4, anti-CD8, and Live/Dead Fixable Aqua, cells were fixed and permeabilised and labelled intracellularly with anti-CD3 and for the presence of IFN-γ, TNF-α, IL-4, and IL-5 as described in our flow cytometric labelling protocol.

### Statistical analyses

Statistical analyses were performed using GraphPad Prism 7 software (La Jolla, CA, USA). Statistical significance was determined by using Mann-Whitney U test, Kruskal-Wallis test, or Two-way ANOVA. For all analyses, *p* values < 0.05 were considered statistically significant.

## Supplementary information

Supplementary Information
