# Supplementary material for: Response kinetics reveal novel features of ageing in murine T cells
Source: Sci Rep. 2019 Apr 3;9:5587. doi: 10.1038/s41598-019-42120-1 (PMC6447543; doi:10.1038/s41598-019-42120-1)
Supplement: Supplementary file 1 — Supplementary Information [file 41598_2019_42120_MOESM1_ESM.docx]

**Response kinetics reveal novel features of ageing in murine T cells**

Daan K.J. Pieren, Noortje A.M. Smits, Martijn D.B. van de Garde, Teun Guichelaar*

Centre for Infectious Disease Control, National Institute for Public Health and the Environment, Bilthoven, The Netherlands

**Supplementary Figures**

**
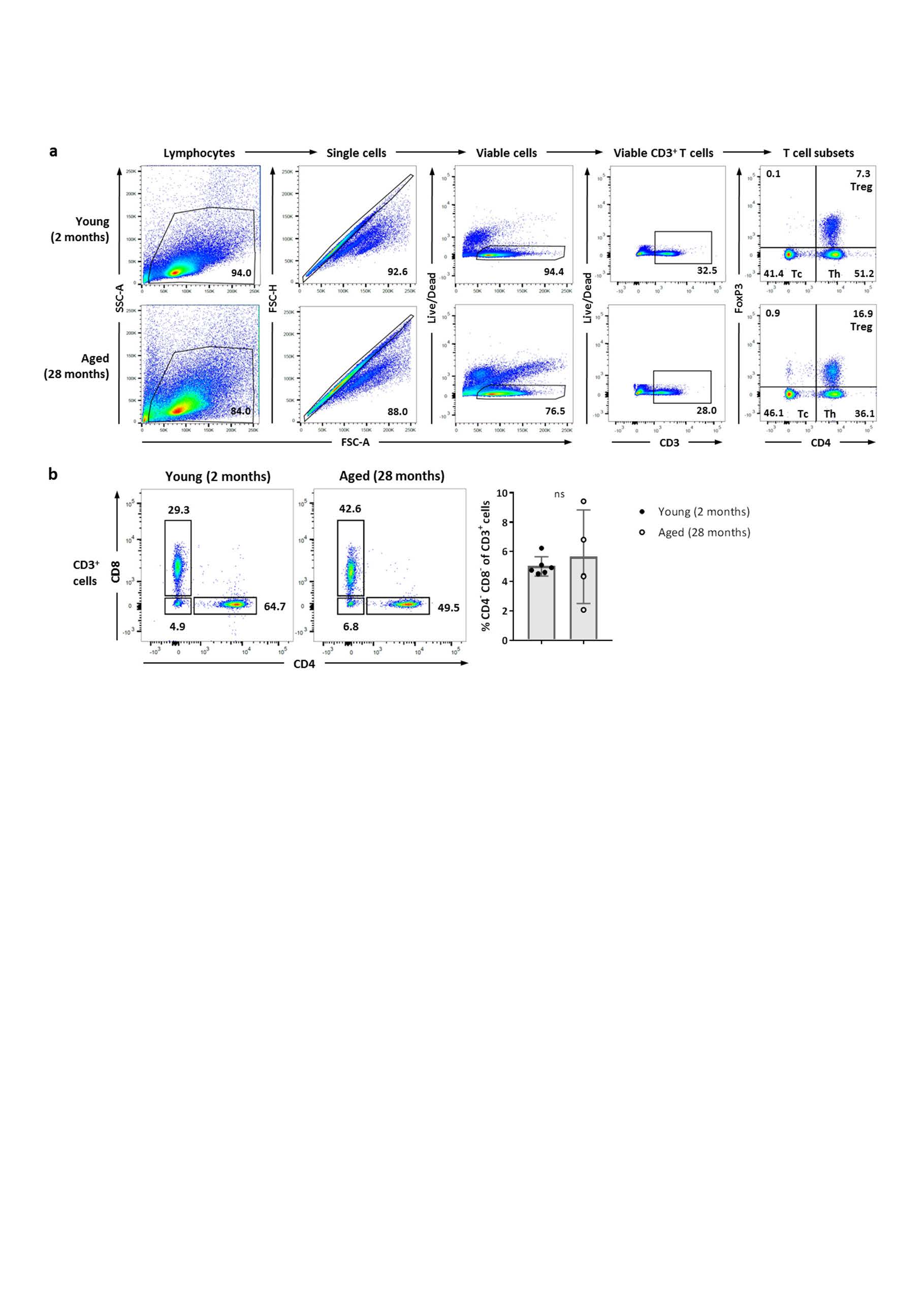
**

**Supplementary Figure 1. Gating strategy towards Th, Tc, and Treg cell subsets and the proportion of CD4^-^CD8^-^ T cells in young and aged mice.**

(**a**) Plots show the gating strategy from splenocyte lymphocytes, single cells, viable cells, viable CD3^+^ cells towards the three T cell subsets; Th cells (FoxP3^-^ CD4^+^ of CD3^+^), Tc cells (FoxP3^-^ CD4^-^ of CD3^+^), and Treg cells (Foxp3^+^ CD4^+^ of CD3^+^) in a young (2 months old) and an aged (28 months old) mouse representing their age group. (**b**) Plots and graphs show the proportion of CD3^+^ T cells that do not express CD4 and CD8 in young and aged mice. Mean ± SD; ns = not statistically significant for difference between young and aged mice using Mann-Whitney test.


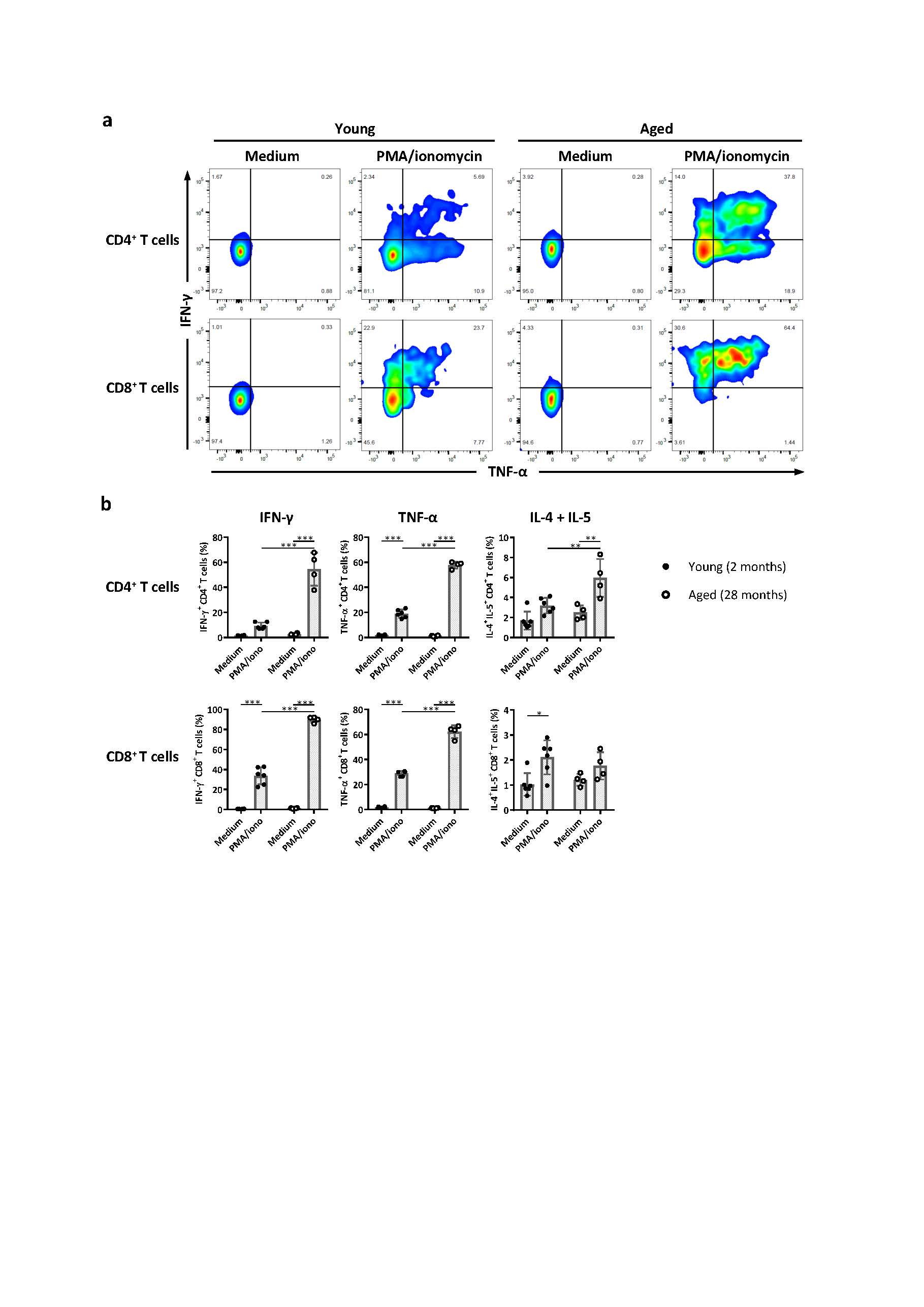


**Supplementary Figure 2. Flow cytometry of intracellular cytokines in CD4^+^ and CD8^+^ T cells of young and aged mice.**

(**a**) Plots show the intracellular expression of IFN-γ and TNF-α in CD4^+^ and CD8^+^ T cells of a young (2 months old) and an aged (28 months old) mouse representative of their age group after stimulation with PMA/ionomycin or medium as unstimulated control for four hours. (**b**) Graphs show the proportions of IFN-γ^+^, TNF-α^+^, or IL-4^+^ + IL-5^+^ in CD4^+^ and CD8^+^ T cells of young (n=6, 2 months old) and aged (n=4, 28 months old) mice. Mean ± SD; **p* < 0.05, ***p* < 0.01, ****p* < 0.001 for difference between young and aged mice using Two-way ANOVA.

**
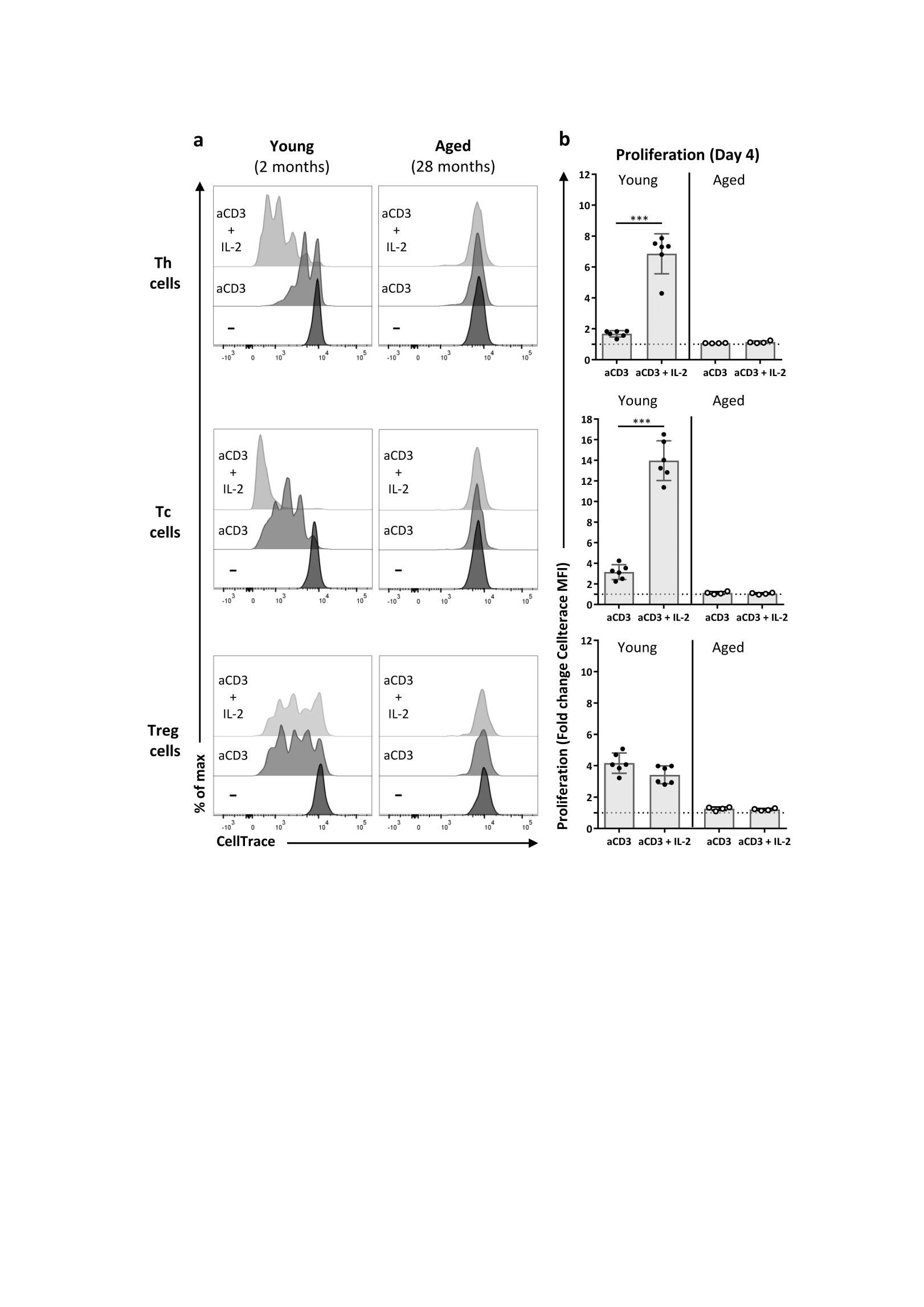
**

**Supplementary Figure 3. T cells of aged mice do not proliferate in response to exogenous IL-2.**

(**a**) Histograms depict the proliferative response of Th, Tc, and Treg cells of young (2 months old) and aged (28 months old) mice measured after four days of exposure to a low anti-CD3 stimulatory strength or low anti-CD3 + exogenous IL-2. Histograms show one mouse that is representative of its age group. (**b**) Graphs show proliferation by fold change in CellTrace MFI of Th, Tc, and Treg cells of young (n=6, 2 months old) and aged (n=6, 28 months old) mice in response to anti-CD3 +/- exogenous IL-2. Mean ± SD; ****p* < 0.001 for difference between young and aged mice using Two-way ANOVA.

**
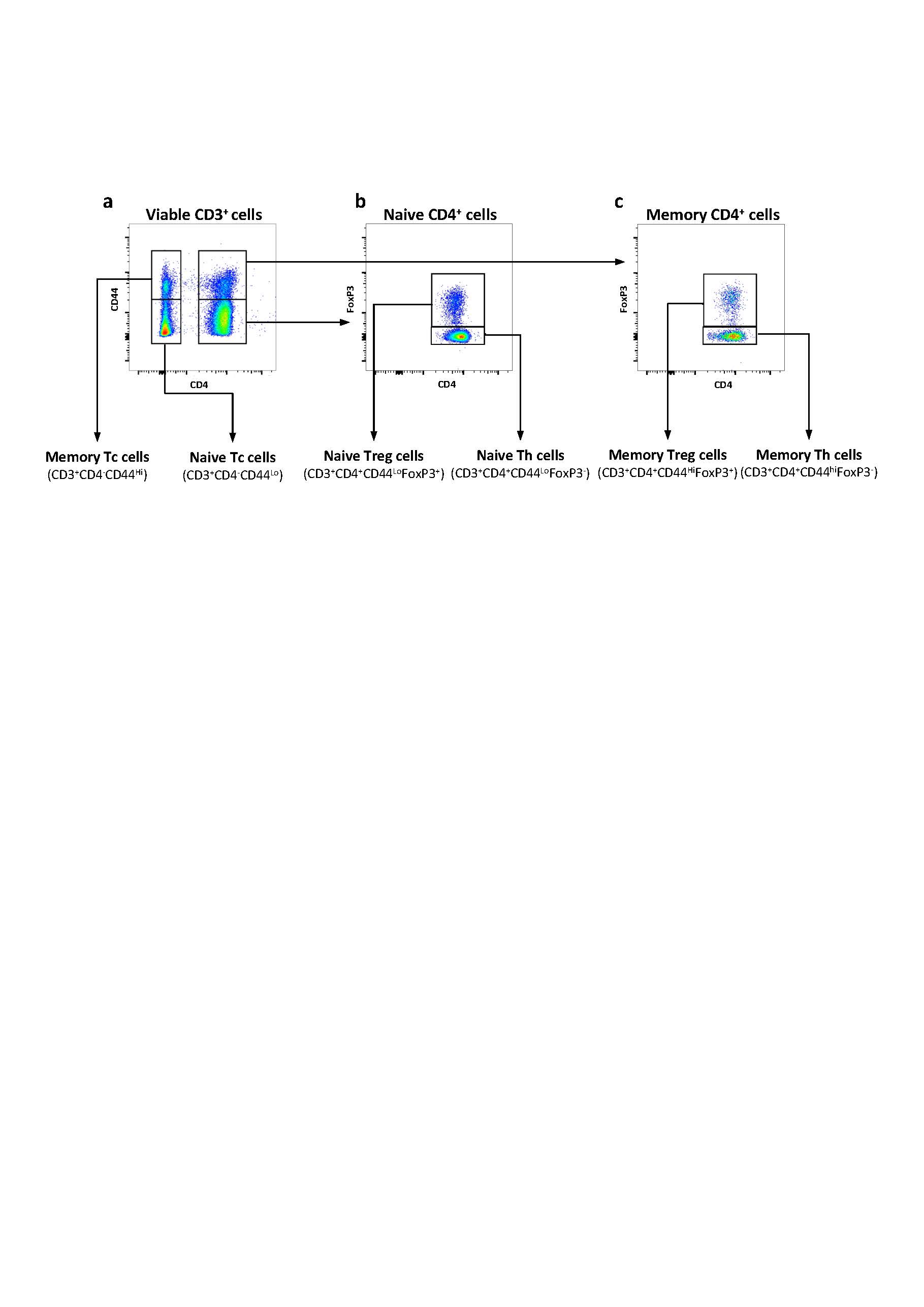
**

**Supplementary Figure 4. Gating strategy towards naive and memory Th, Tc, and Treg cell subsets for viSNE analyses.**

For our viSNE analysis we gated on naive and memory T cell subsets based on CD44 expression, here shown for one young mouse. Viable CD3^+^ T cells were gated on (**a**) naive Tc cells (CD3^+^CD4^-^CD44^Lo^), memory Tc cells (CD3^+^CD4^-^CD44^Hi^), naive CD4^+^ T cells (CD3^+^CD4^+^CD44^Lo^), and memory CD4^+^ T cells (CD3^+^CD4^+^CD44^Hi^). On these four subsets, we performed the viSNE analyses shown in Figure 5. Based on the viSNE analyses, we further divided (**b**) naive CD4^+^ T cells (CD3^+^CD4^+^CD44^Lo^) and (**c**) memory CD4^+^ T cells (CD3^+^CD4^+^CD44^Hi^) into naive Treg cells (CD3^+^CD4^+^CD44^Lo^FoxP3^+^), memory Treg cells (CD3^+^CD4^+^CD44^Hi^FoxP3^+^), naive Th cells (CD3^+^CD4^+^CD44^Lo^FoxP3^-^), and memory Th cells (CD3^+^CD4^+^CD44^Hi^FoxP3^-^).


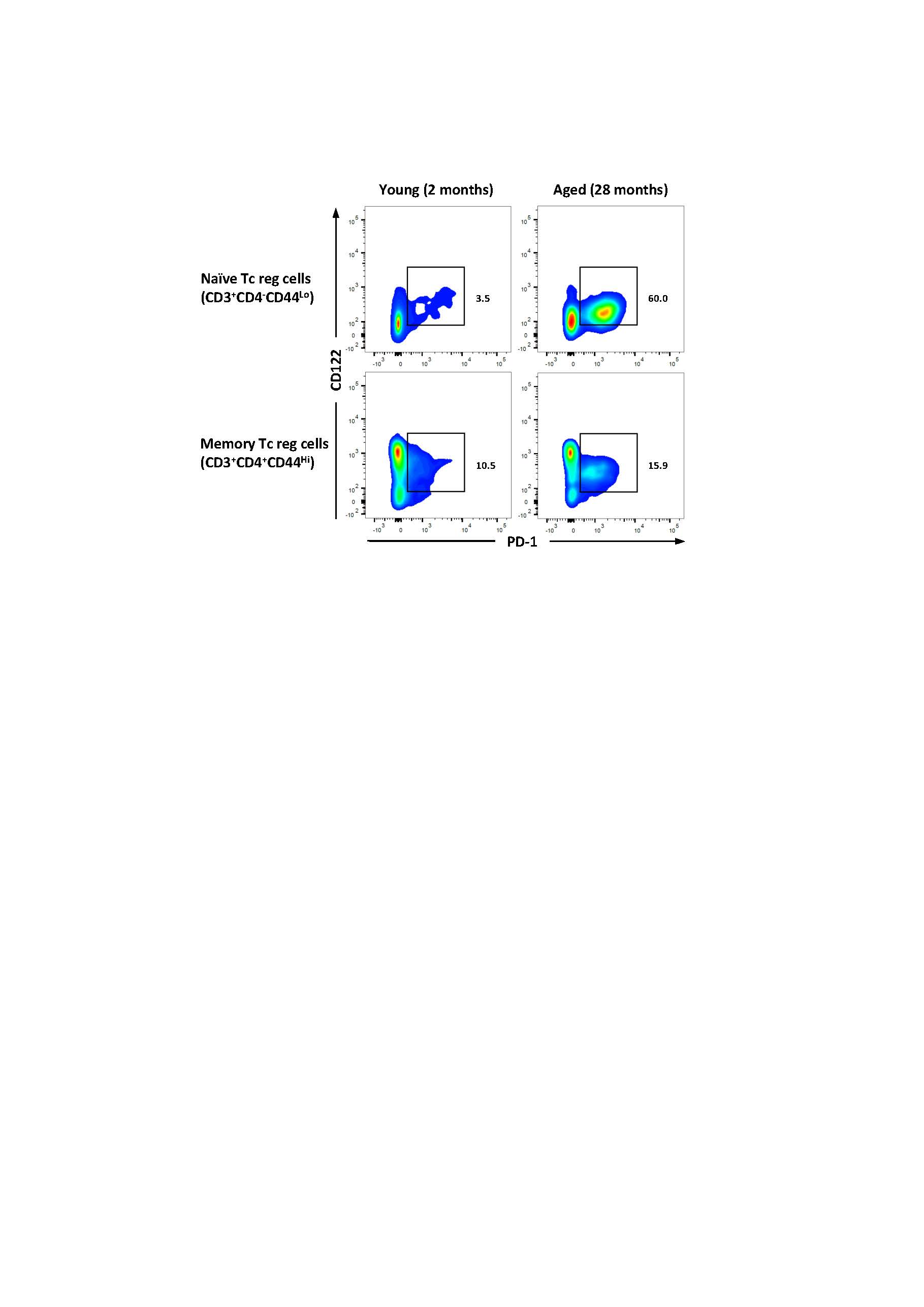


**Supplementary Figure 5. Gating strategy of regulatory Tc cells.**

Plots show gating of naive and memory Tc reg cells by expression of CD122 and PD-1 in a young (2 months old) and an aged (28 months old) mouse that represent their age group. Based on simultaneous expression of CD122 and PD-1 to identify Tc reg as previously reported, naive Tc reg cells were defined as CD3^+^CD4^-^CD44^Lo^CD122^+^PD-1^+^ and memory Tc reg cells were defined as CD3^+^CD4^-^CD44^Hi^CD122^+^PD-1^+^.


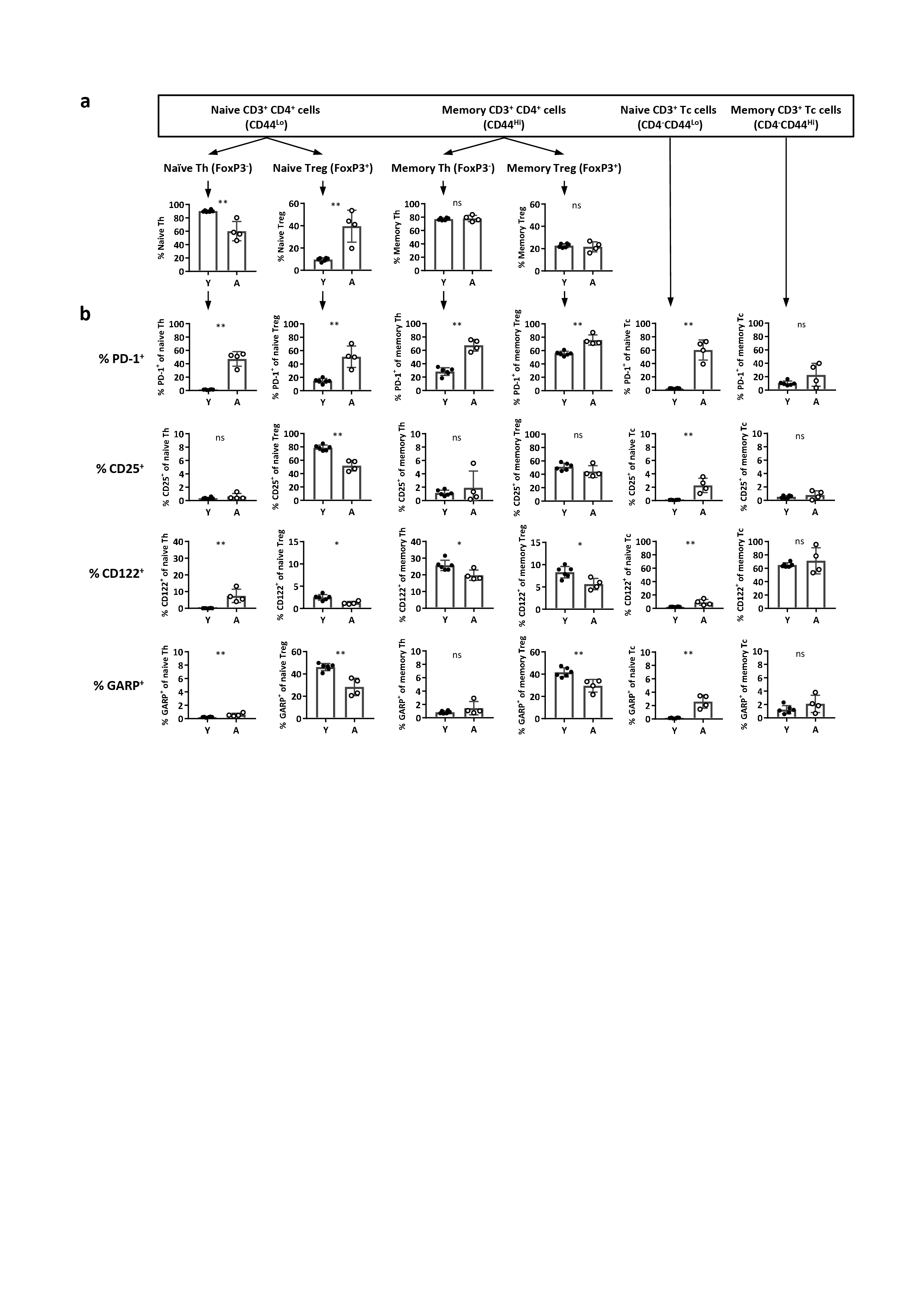


**Supplementary Figure 6. Frequencies of PD-1^+^, CD25^+^, CD122^+^, and GARP^+^ naive and memory T cell subsets.**

Graphs show the frequencies of PD-1^+^, CD25^+^, CD122^+^, and GARP^+^ T-cell subsets used to verify results generated by viSNE. (**a**) Upper (boxed) naive and memory T cell subsets are those that have been analysed by viSNE. Based on FoxP3 expression, naive CD3^+^CD4^+^ cells were subdivided into naive Th cells (CD44^Lo^Foxp3^-^) and naive Treg cells (CD44^Lo^Foxp3^+^), and memory CD3^+^CD4^+^ cells were subdivided into memory Th cells (CD44^Hi^Foxp3^-^) and memory Treg cells (CD44^Hi^Foxp3^+^). (**b**) Frequencies of PD-1^+^, CD25^+^, CD122^+^, and GARP^+^ cells were determined in the naive and memory subsets of Th, Treg, and Tc cells in spleens from young (n=6, 2 months old) and aged (n=4, 28 months old) mice. Mean ± SD; **p* < 0.05, ***p* < 0.01, ns = not statistically significant for difference between young and aged mice using Mann-Whitney test.


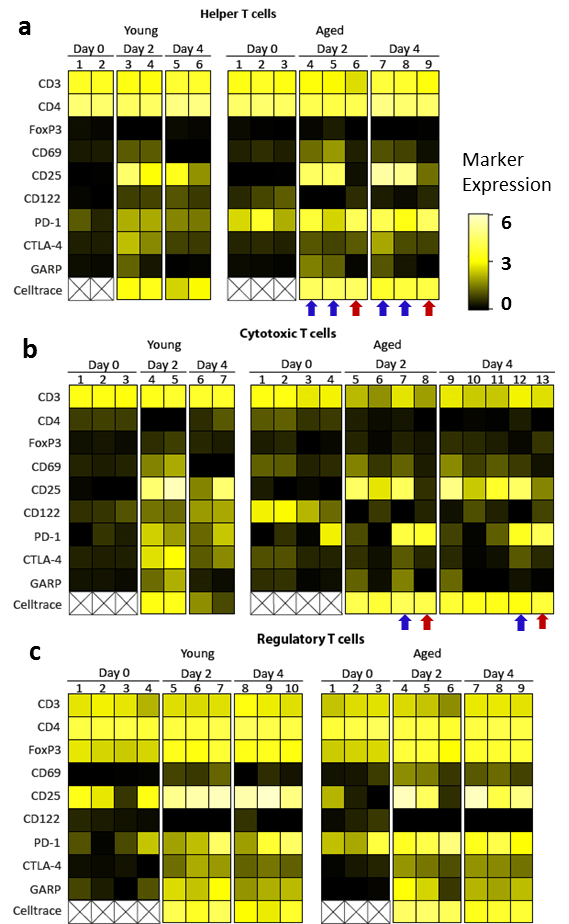


**Supplementary Figure 7. Heatmaps of indicated markers included in viSNE analyses performed on Th, Tc, and Treg cells of young and aged mice before and after stimulation.**

Expression of T cell markers before and after exposure of splenocytes of young (n=6, 2 months old) and aged (n=4, 28 months old) mice to an intermediate stimulatory strength for two and four days. Heatmaps depict the ArcSinh5-transformed median expression of the designated markers in Th (**a**), Tc (**b**), and Treg (**c**) cell clusters at the indicated days of young and aged mice. Numbers above each heatmap correspond to the cluster numbers identified by viSNE as shown in Figure 6. Red arrows indicate the non-activated (CD25^-^CD69^-^) PD-1^+^ Th and Tc cell clusters in aged mice (**a,b**). Blue arrows indicate the activated (CD25^+^CD69^+^) PD-1^+^ Th and Tc cell clusters in aged mice (**a,b**).


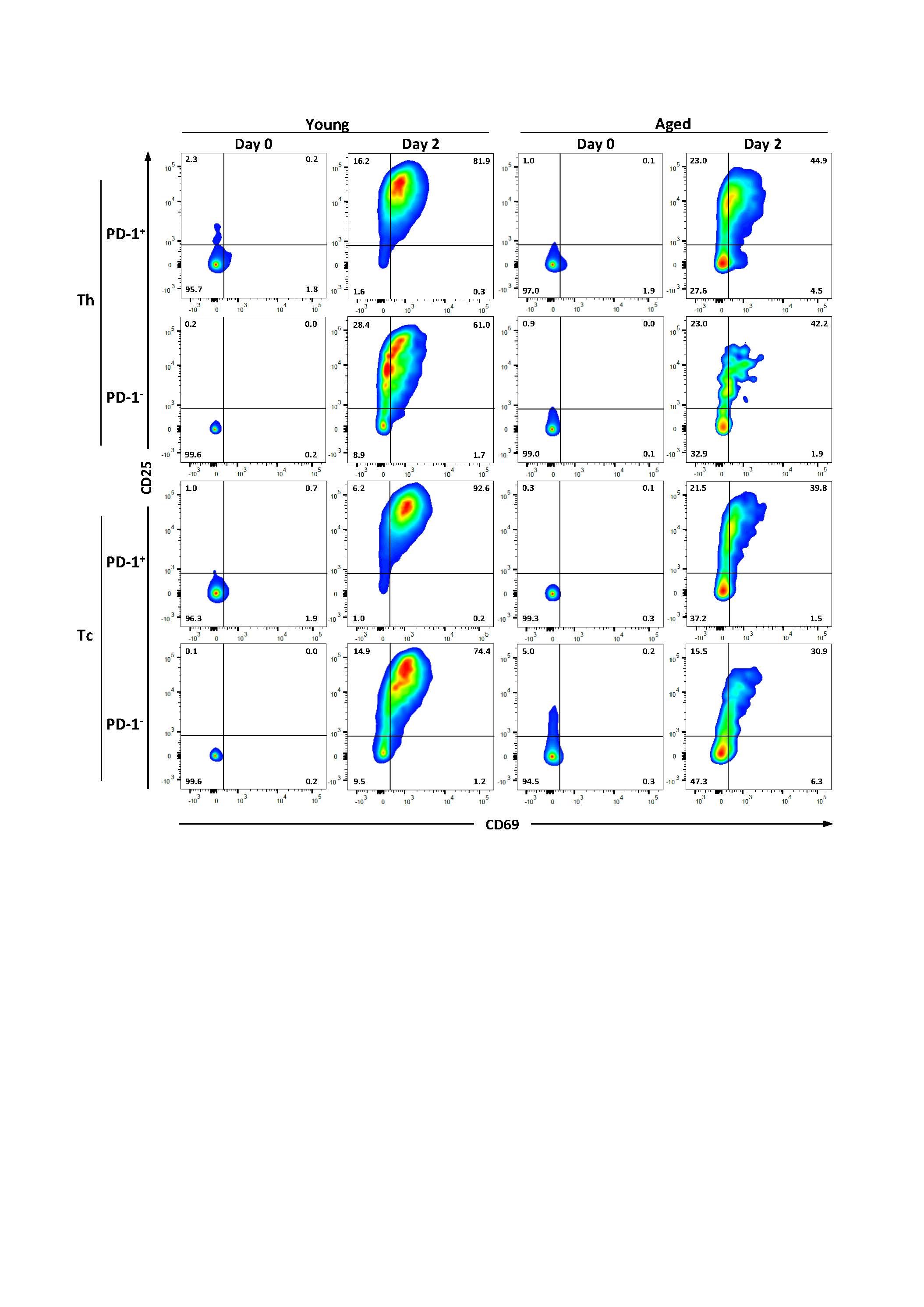


**Supplementary Figure 8. Gating of PD-1^+^ or PD-1^-^ Th and Tc cells expressing CD25^+^ and CD69^+^ before and after stimulation.**

Plots show the expression of CD25 and CD69 in PD-1^+^ and PD-1^-^ (**a**) Th and (**b**) Tc cells before and after two day exposure to an intermediate stimulatory strength. Plots depict analyses of one young (2 months old) and one aged (28 months old) mouse that represent their age group.
